# Immunoprophylaxis of respiratory syncytial virus: global experience

**DOI:** 10.1186/rr187

**Published:** 2002-06-24

**Authors:** Eric AF Simoes

**Affiliations:** 1 Department of Pediatrics, Section of Infectious Diseases, The University of Colorado School of Medicine and The Children's Hospital, Denver, Colorado, USA

**Keywords:** monoclonal antibody, palivizumab, passive immunity, respiratory syncytial virus, Synagis^®^

## Abstract

Respiratory syncytial virus (RSV) infects nearly all children by age 2 years, and it causes considerable illness and death in certain high-risk pediatric populations. Historically, treatment for RSV has been symptomatic, and developing a safe and effective vaccine has been a challenge. Therefore, research efforts have turned to passive immunization as the best option to control RSV. Palivizumab, a genetically engineered humanized monoclonal antibody, has been shown to reduce RSV-related hospitalizations significantly, with few adverse effects. It was approved for use in high-risk children in the USA in 1998 and in Europe in 1999; it is now approved for use in more than 45 countries. The efficacy and safety of palivizumab continue to be supported by both clinical trial and outcomes data.

## Introduction

Respiratory syncytial virus (RSV) infection in previously healthy children usually results in mild upper respiratory tract disease that resolves spontaneously, but in a minority (usually infants <3 months old) it causes serious disease. In certain high-risk pediatric groups, however, severe lower respiratory tract infection (LRTI) with considerable illness and death is a more frequent occurrence. These groups include preterm infants with and without chronic lung disease (CLD), children with pulmonary or cardiac disease, and immunocompromised children. Potential long-term consequences of LRTI caused by RSV include possible associations with (and future development of) asthma, allergies, and other chronic pulmonary illnesses. Some studies suggest that severe RSV LRTI is associated with recurrent wheezing, asthma, and other pulmonary sequelae [1-3].

Infection with RSV has far-reaching effects, with both the US Centers for Disease Control and Prevention [4] and the World Health Organization [5] reporting that RSV is the leading cause of LRTI among infants and children worldwide. Low income, poor nutrition, and low birth weight are additional risk factors that are particularly important in developing countries, where it has been estimated that approximately four million children die each year from acute respiratory tract infections [6]. An estimated 100,000–125,000 hospitalizations and 100–450 deaths annually among infants and children in the USA are attributed to RSV LRTI [7-9]. The number of children hospitalized for bronchiolitis has also increased markedly in the USA. From 1980 to 1996, hospitalization rates per 1000 children under 1 year old increased from 12.9 to 31.2 [7]. In most patients hospitalized for bronchiolitis (50–90%) and in up to half of those for winter pneumonia (20–50%), RSV is the underlying cause [10].

Reinfection is common in older children, adults, and the elderly because, unlike a number of other infectious diseases, infection with RSV does not confer protective and enduring immunity. Therefore, individuals at high-risk (e.g. those who are debilitated or geriatric patients, patients with chronic heart or lung disease, or immunosuppressed individuals) are also at risk for morbidity from the complications of RSV.

Research for effective treatment strategies for RSV has been ongoing for almost four decades with few successes. Development of an effective RSV vaccine has many challenges, including the need to induce immunity to the multiple strains of RSV. Also, because the children at highest risk are younger than 3 months, vaccination would ideally take place when the child is still a newborn (≤ 1 month old), raising concerns regarding possible interference by maternal antibodies to injectable vaccines. A further concern is that because natural infection does not prevent reinfection, a series of boosters may be needed. Finally, the experience of enhanced pathology following administration of a formalin-inactivated vaccine to naïve infants in the 1960s upon subsequent RSV infection [11,12] has tempered the development of injectable vaccines for RSV. Various animal models have been developed to study the enhanced pathology seen in these infants, such as the cotton rat [13], mouse [14,15], and monkey [16,17]. Live, attenuated RSV vaccines showed promise in early trials because they did not result in more severe disease after later natural infection with RSV, could be given intranasally, and protected against RSV-induced upper respiratory tract infections and LRTIs [18]. Like with other live vaccines, however, vaccinees have the potential to shed virus, and this live RSV vaccine has not been sufficiently attenuated. A live, genetically engineered RSV vaccine is a future prospect [19,20]. Subunit vaccines that contain purified F and G glycoproteins or novel chimeric substances splicing together F and G proteins are currently being studied [21], as are DNA vaccines [22]. However, current vaccination strategies are focused on maternal immunization and live vaccines.

## Passive immunoprophylaxis: immunoglobulins

During the 1980s passive immunoprophylaxis was studied as an alternative to the live vaccines, which had failed to provide acceptable protection against RSV infection. Early studies in rats, as well as clinical observations in infants infected with RSV, demonstrated that titers of RSV antibodies needed to be between 1:200 and 1:400 to prevent LRTI [23,24]. Standard immunoglobulin preparations did not adequately protect the lower respiratory tract against RSV infection, seemingly because of the low titers that could be achieved.

### Introduction of respiratory syncytial virus immune globulin

RSV immune globulin (RSV-IGIV; RespiGam™, MedImmune Inc., Gaithersburg, MD, USA) contains a sixfold higher concentration of RSV neutralizing antibodies than do standard immunoglobulin preparations. It was developed to improve on the record of standard immunoglobulin preparations, and specifically to provide passive immunity against RSV in infants who were born preterm, before the third trimester when maternal IgG antibodies are typically passed from the mother to the fetus [25,26].

A 3-year, prospective, randomized, blinded, multicenter study was conducted between 1989 and 1992 that compared results after use of RSV-IGIV at doses of either 750 or 150 mg/kg with those in control individuals who did not receive RSV-IGIV [23]. In the 249 infants and children born prematurely, or with bronchopulmonary dysplasia (BPD) or congenital heart disease (mean age 8 months), those treated with the higher dose had a significantly lower rate of LRTI, fewer and shorter hospitalizations, fewer admissions to the intensive care unit, and required less of the antiviral agent ribavirin than did control individuals. During the 1994–1995 RSV season, these results were replicated in a larger multicenter study in the US-based PREVENT study of 510 infants and children born prematurely or with BPD [24]. In that randomized, double-blind, placebo-controlled study, hospitalization was reduced by 41% and RSV-IGIV was found to be safe and well tolerated.

### Limitations of respiratory syncytial virus immune globulin

Although RSV-IGIV offered distinct advantages over the formalin-inactivated vaccine (primarily protection of the lower respiratory tract without more severe infection after later challenge with wild-type virus), it also has various limitations. For example, it is not effective in children with congenital heart disease or cyanotic heart disease because of hyperviscosity, and was responsible for more hypercyanotic events than was albumin [27]. In addition, administration of RSV-IGIV is time-consuming and inconvenient, involving 3- to 4-hour intravenous infusions of large fluid volumes and protein loads each month. This can lead to fluid overload in some children [23,24] and is of special concern when children, particularly infants, have chronic cardiopulmonary conditions.

### Monoclonal antibodies

Monoclonal antibodies were investigated in an effort to avoid the difficulties associated with RSV-IGIV. The first monoclonal preparations could be administered intranasally, thereby protecting the portal of entry and precluding the difficulties associated with parenteral therapy.

In a study using rhesus monkeys [28], a mouse monoclonal IgA antibody against RSV F glycoprotein was administered as nose drops. The monkeys developed high titers of RSV neutralizing antibodies, but this finding was not repeated in human phase III clinical trials and efficacy could not be proved. In addition, because the half-life of IgA is short, dosing schedules would require repeated applications, hence reducing the likelihood of compliance.

Likewise, a clinical trial with the intramuscular IgG humanized monoclonal antibody SB 209763 also failed to produce favorable results [29,30]. The development of a humanized monoclonal antibody produced by recombinant DNA technology – palivizumab – represented a major advance in protection against RSV.

## Experience with palivizumab

Palivizumab (Synagis^®^; MedImmune Inc., Gaithersburg, MD, USA) is a humanized monoclonal antibody (IgG_1_) that is directed at an epitope on the A domain of the F glycoprotein on the surface of the RSV. Its mechanism of action is to neutralize and inhibit the fusion activity of both types A and B clinical RSV isolates on respiratory epithelial cells [31]. Unlike RSV-IGIV, palivizumab is not derived from human blood and does not require intravenous administration. Its greater safety and convenience of use are clear advantages over previous methods of passive immunoprophylaxis. It is administered seasonally to high-risk individuals by monthly intramuscular injections.

### The IMpact-RSV study

The IMpact-RSV study was the basis for approval of palivizumab by the US Food and Drug Administration and by the European Agency for the Evaluation of Medicinal Products. That randomized, double-blind, placebo-controlled study (conducted between 1996 and 1997) demonstrated that palivizumab could safely and effectively be administered to children who are at high risk for serious LRTI associated with RSV. Patients involved in the study were from 139 centers across the USA, the UK, and Canada. Each of 1502 children born prematurely and/or with BPD received either five monthly injections of palivizumab intramuscularly (15 mg/kg) or treatment with an equivalent dose of placebo. The children were monitored for hospitalization rates, LRTI, duration of hospital stay, and use of supplemental oxygen and/or mechanical ventilation or other intensive care procedures [32].

Adverse events were infrequent, and 99% of children in both the palivizumab and placebo groups completed the treatment protocol [32]. Overall hospitalization rates for RSV infection were reduced by 55% in children who received palivizumab (4.8%) as compared with rates in children who received placebo (10.6%; *P* < 0.001). Hospitalization rates were reduced by 78% in children without BPD and by 39% in children with BPD. Among the latter group of children, hospitalization rates were 7.9% for those who received palivizumab versus 12.8% for those who received placebo. Finally, hospitalization rates for preterm infants were 1.8% in those who received palivizumab versus 8.1% in those who received placebo.

In addition to being the pivotal study for safety and efficacy of palivizumab, the IMpact-RSV study served as the basis for the usage guidelines of the American Academy of Pediatrics (AAP) [33] and the European consensus guidelines [34]. The AAP recommended the following criteria be used to determine eligibility for prophylaxis with palivizumab [33]: infants born at ≤ 28 weeks of gestation and who are under 1 year old at the start of the RSV season; infants born at 29–32 weeks of gestation and who are under 6 months old at the start of RSV season; infants born at 32–35 weeks of gestation, who are under 6 months old at the start of RSV season, and are at higher than average risk for RSV exposure (e.g. through day-care attendance or school-age siblings); and children under 2 years old who have a CLD requiring medical management in the last 6 months.

### Initial field experience

During the first RSV season (1998–1999) after approval in the USA, palivizumab was administered to approximately 56,000 patients. Results of the initial field experience analyzed through a retrospective review of the charts of 1839 patients from nine sites in the USA [35] were consistent with those obtained in the IMpact-RSV study. The hospitalization rate for RSV infection in patients who received palivizumab was 2.3%, with only 42 out of the 1839 patients requiring hospital admission. Hospitalization rates were 4.0% (16 out of 399) for patients with CLD, and 2.1% (26 out of 1227) for premature infants without CLD.

During the second season of palivizumab use (1999–2000) field experience was again evaluated by means of a retrospective chart review, this time in 2830 children from 12 sites in the USA [36]. Again, results were consistent with those of the IMpact-RSV study. The hospitalization rate for RSV-related illness was 2.4% (68 out of 2830) in children who received palivizumab. Rates were 3.9% (31 out of 795) in children with CLD and 1.3% (34 out of 2542) in otherwise healthy but preterm infants [36].

During the third season (2000–2001), experience with palivizumab was evaluated via use of the Synagis Outcomes Registry [37]. This prospective, multicenter effort involved 2095 children given palivizumab according to the monthly dosing protocol at 62 pediatric offices and clinics across the USA. The data collected added to the favorable profile of palivizumab administration that had already been observed in the IMpact-RSV study and the previous retrospective investigations. The infants involved in the Synagis Outcomes Registry were mostly Caucasian, with 947 considered high-risk because their gestational age was between 32 and 35 weeks and, in 66% of the group, because of one or more additional risk factors. These risk factors included multiple birth (32%), CLD (24%), child care by either the child or a sibling (22%), exposure to tobacco smoke (16%), congenital heart disease (5%), and cystic fibrosis (0.6%). In that investigation, hospitalization rates were 2.9% overall, 5.8% in infants with CLD, and 2.1% in premature infants without CLD.

### Global experience

A phase III/IV multicenter, single-arm, open-label, expanded access study was performed between November 1998 and March 1999 in 15 countries in the northern hemisphere because palivizumab was not approved for use in all countries [38]. Children were included in the study based on the AAP guidelines discussed above. Palivizumab was administered intramuscularly to each child at a dosage of 15 mg/kg, according to the monthly dosing protocol. The children were monitored for adverse events for 150 days, during which time 40 drug-related adverse events were reported in 39 children (6.9%). All of the reported adverse events were mild or moderate, with injection site reactions being most common (12 out of 530). Fever was reported in eight children, and diarrhea and irritability in four. Two children died, but the deaths were unrelated to either RSV or receiving palivizumab. In approximately 25% of the children tested for RSV (7 out of 29), RSV was the cause of hospitalization. Considering this rate to be a reasonable assessment of RSV infection among children who were not actually tested, the estimated overall hospitalization rate was 2.1%. The low incidence of adverse events and low hospitalization rates reported support earlier studies of the safety, tolerability, and effectiveness of palivizumab.

In France palivizumab was made available to high-risk children before approval by the European Agency for the Evaluation of Medicinal Products by permission of the French Agency for the Safety of Health Products. Because this was an observational field survey, there was no control group, blinding, or randomization (unpublished data). Data were collected on 516 preterm infants (median gestational age 28 weeks, with 88% born at or before 32 weeks) who received one to five monthly injections of palivizumab. The rate of BPD was 81%, which is higher than in the IMpact-RSV and PREVENT studies. The hospitalization rate for RSV disease was 7.6% (*n* = 39), with 10 infants requiring intensive care and four requiring mechanical ventilation. There were no deaths attributed to administration of palivizumab.

In Canada, a special access program was instituted to allow use of palivizumab in high-risk children before its approval by Canadian regulatory authorities. Use of palivizumab and compliance with dosing schedules were monitored in the COMPOSS study (Compliance, Outcomes, Management: a Prospective, Observational Study of Synagis in Canada) [39], which was conducted during the 1999–2000 season. Among the 480 infants who were born prematurely and/or had BPD, compliance was high; 77% of the 1700 doses given were administered according to the recommended schedule, and only two infants were withdrawn from the program because of perceived adverse effects. Hospitalization rates for RSV were even lower than those in other studies, at 2.4% overall (1.6% in premature babies and 6.0% in infants with BPD). This overall rate was half that seen in the IMpact-RSV study (4.8%), possibly because the latter study included more children with BPD. COMPOSS did not have a control group, and therefore direct comparison with the IMpact-RSV study is not possible. Nevertheless, hospitalization rates due to RSV in the placebo groups of both the PREVENT and IMpact-RSV studies were substantially higher than those in COMPOSS.

In The Netherlands, a retrospective analysis was conducted in 450 children who received palivizumab during the 1999–2000 season [40]. The children involved were either born preterm, or had chronic pulmonary and/or cardiac disease, and/or immunodeficiency. Evaluation forms were completed by the children's pediatricians (70% response rate), and data were collected on sex, date of birth, gestational age, risk factors for serious RSV infection, and hospitalization rates. Of the children who received palivizumab, only 1.6% overall (4 out of 254) were hospitalized for RSV infections; in infants born at 29–32 weeks of gestation, hospitalization rates were 0.8% in those without CLD and 6.7% in those with CLD.

Additional data on the safety and tolerability of palivizumab were collected in the PROTECT (Palivizumab RSV Open-label Trial of Effectiveness and Clinical Tolerability) study [41]. In that study, which was conducted in 35 centers in 17 countries, the preterm infants (born at gestational age between 29 and 32 weeks) included differed from those in other studies in that they did not have pulmonary disease. In children 6 months old or younger (*n* = 285) who received palivizumab monthly at a dosage of 15 mg/kg intramuscularly, the most commonly reported adverse events were cough, rhinitis, pharyngitis, bronchiolitis, fever, and diarrhea. Twenty children were hospitalized for respiratory infections; six tested positive for RSV and 14 tested negative. The rate of hospitalization due to RSV was 2.1%.

### Continued experience in Europe

Because palivizumab is partly murine derived, there is a theoretical, although unlikely, possibility that a person could mount an immune response (e.g. antibody formation, or allergic or anaphylactic responses) to these components on repeated exposure. A study conducted to investigate potential immunologic events, as well as to continue to collect data on safety and efficacy, was conducted in 14 centers in Europe and Canada [42]. A total of 134 children were included; palivizumab was administered to 71 of the children for the first time and to 63 of the children for their second season. No immune reactions, including production of antipalivizumab antibodies, or other serious adverse events were observed. Mean serum trough levels of palivizumab were greater than 30–40 g/ml in all children, which is adequate for protection against RSV. The hospitalization rate due to RSV infection was 4.2% overall.

Because of the previous problems of increased disease severity after challenge with wild-type virus associated with the formalin-inactivated vaccine, a phase III/IV cohort follow-up study was conducted [43]. In that study, data collected from children in Europe and Canada showed no difference between treated and control groups in overall hospitalization rates or occurrence of adverse events. The treated group consisted of children who had received at least two doses of palivizumab during the previous RSV season; the control group consisted of children who had never received palivizumab. The two groups were matched in terms of sex and gestational age. The study showed that, unlike the old formalin-inactivated vaccine, palivizumab does not cause more frequent or severe RSV disease.

### Combined analysis of hospitalization rate data

**Figure 1:**
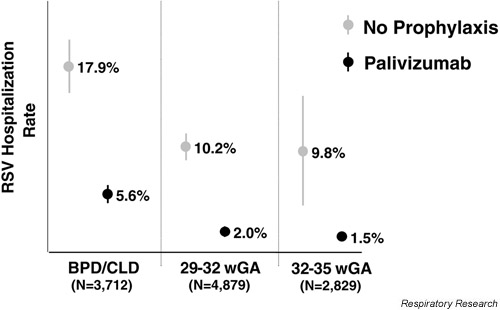
Weighted mean hospitalization rates due to RSV (standard deviation indicated by vertical bars) in children ≤ 2 years old with BPD/CLD, in infants at 29–32 weeks' gestational age (wGA) without CLD, and in infants at 32–35 wGA without CLD (combined analysis). Data from Simoes [44].

A meta-analysis of RSV hospitalization in various risk groups is summarized in Table 1 and Fig. 1. Hospitalization rates were examined in both prophylaxed and unprophylaxed children in a combined analysis of the data from numerous studies [44]. The studies included were either prospective or retrospective, and either controlled trials or not. However, in all studies the following had to apply: hospitalization rates had to have been defined as the total number of patients hospitalized due to RSV at least once divided by the total number of patients in the study; and subpopulations had to have been clearly defined. The three subpopulations examined were children younger than 2 years old with BPD/CLD, infants at gestational age 29–32 weeks without CLD, and infants at gestational age 32–35 weeks without CLD. In each subpopulation, hospitalization rates were considerably lower in patients who had been treated with palivizumab than in those who had not. In the BPD/CLD children, rates were 17.9% and 5.6% for those who received palivizumab and did not receive palivizumab, respectively. In the infants at gestational age 29–32 weeks the corresponding rates were 10.2% and 2.0%, and in the infants at gestational age 32–35 weeks the rates were 9.8% and 1.5%.

### Special uses

Many children who do not meet the official AAP criteria for eligibility to receive palivizumab [33] may still benefit from and may be considered for treatment. In one study [45] palivizumab was administered to 86 children who did not meet the AAP criteria but had BPD when they were aged 2 years or older, or had other chronic illnesses including cerebral palsy, trisomy 21, chronic aspiration, severe tracheomalacia, cystic fibrosis, or myotonic dystrophy. No adverse events or deaths were reported, and the hospitalization rate was 3.5%, although the rate in populations without prophylaxis is unknown.

Palivizumab has been used as prophylaxis during nosocomial outbreaks. Because RSV is ubiquitous in the environment, can be shed for up to 4 weeks in newborns, and can remain on fomites for up to 7 hours and on skin for 30 min [46], it causes nosocomial outbreaks in nurseries and intensive care units. Until the introduction of palivizumab, the only mechanisms to control RSV nosocomial outbreaks were standard infection control techniques, including handwashing, minimizing contact between hospital personnel and infected patients, using gowns and gloves, and disinfecting processes. Although use of palivizumab in such situations is off-label, it has been used in several hospitals in Europe to protect infants against nosocomial RSV infection.

Such nosocomial outbreaks have occurred and been controlled with use of palivizumab in the UK, Spain, and Portugal. In Northamptonshire, UK (November–December 1999), seven preterm infants in a special care unit developed RSV infections [47]. One of the infants developed severe bronchiolitis and pneumonia that resulted in death. Eight other high-risk infants who were administered palivizumab at 15 mg/kg did not develop RSV infection.

In Barcelona, Spain (1999), similar results were observed in a larger study involving 52 neonates during an outbreak of RSV with four confirmed cases [48]. All of the infants received palivizumab. Three out of the four infants who had confirmed RSV infections were tested and were found to be free of RSV 6 days after administration of palivizumab. None of the other 48 neonates developed RSV infection, with no adverse events. No deaths were reported and mechanical ventilation was not needed. A similar outbreak that had occurred in that same care facility 11 years previously – using the same infection control techniques but before palivizumab was available – resulted in an RSV infection rate of 32% and an inability to accept new admissions into the facility for 7 weeks.

In Portugal (November–December 1998), two outbreaks of RSV occurred 2–3 months apart in a neonatal unit caring for 19 infants (data on file, Abbott Laboratories). Despite implementation of standard infection control techniques after three neonates developed RSV infection, infection developed in eight more of the neonates. One of the infants died and three others needed respiratory support via mechanical ventilation. After all neonates received palivizumab, no further RSV infections developed and the outbreak was stopped.

Although none of those studies was controlled, the results suggest that administration of palivizumab may be useful in preventing nosocomial spread of RSV. Additional research is warranted for confirmation.

### Continued field monitoring of palivizumab

A favorable safety profile has been documented over the four seasons of use of palivizumab. Pharmacovigilance measures are in place to continue to confirm the safety profile. Such measures increase the possibility of identifying any serious or infrequent adverse events through reporting of such events in larger and more varied patient groups under conditions of field use.

Between October 1998 and June 2001, a total of 272,879 patient exposures occurred (data on file, Abbott Laboratories). When the incidence of adverse events was compared with that in the IMpact-RSV trial, no increase was seen. Urticaria was seldom reported (20 out of 125,546 exposures [0.016%]). The overall frequency of death was not greater in patients who received palivizumab, and none of the 121 deaths reported were related to administration of palivizumab.

More than five monthly palivizumab injections may be required in countries with an extended RSV season. Safety was assessed in this situation by examining the records of 433 infants who had received six or more treatments with palivizumab [49]. In those infants the rate of hospitalization due to RSV was 2.1% and the rate of adverse events was 1.6%. These findings compare favorably with those of the IMpact-RSV study, in which no more than five treatments were given.

## Conclusion

Palivizumab is a humanized monoclonal antibody that is administered via monthly injections and confers passive immunity against development of severe RSV disease in high-risk children. Multiple studies and postmarketing experience involving thousands of preterm and high-risk infants in various demographic groups have demonstrated that treatment with palivizumab decreases overall rates of hospitalization due to RSV. Palivizumab enjoys an excellent overall safety profile, as confirmed by the low incidence of adverse events in multiple studies and in phase IV testing conducted since its first season of use.

Thus far, the search for a safe and effective vaccine against RSV has not succeeded, and clinical outcomes in studies of children treated symptomatically for RSV with bronchodilators, steroids, and antiviral agents (ribivarin) have not been improved [50,51]. Until such a vaccine is discovered and proven, palivizumab remains the only safe, effective, and convenient treatment to prevent RSV disease in young children who are at risk.

## Abbreviations

AAP = American Academy of Pediatrics; BPD = bronchopulmonary dysplasia; CLD = chronic lung disease; LRTI = lower respiratory tract infection; RSV = respiratory syncytial virus; RSV-IGIV = respiratory syncytial virus immune globulin.

## Figures and Tables

**Table 1:** Hospitalization rates

	Children ≤ 2 years old with BPD/CLD (*n* = 3675)		Infants at gestational age 29–32 weeks without CLD (*n* = 4854)		Infants at gestational age 32–35 weeks without CLD (*n* = 2829)	
			
Reference (country)	Untreated	Palivizumab	Untreated	Palivizumab	Untreated	Palivizumab
[23] (USA)	36.7% (30)	-	-	-	-	-
[24] (USA)	17.4% (149)	-	-	-	-	-
[52] (USA)	25.2% (131)	-	7.6% (662)	-	-	-
[53] (UK)	19.1% (235)	-	-	-	-	-
[32] (USA/Can/UK)	12.8% (266)	7.9% (496)	8.5% (142)	1.6% (313)	9.8% (123)	2.0% (250)
[35] (USA; outcomes 1998–1999)	-	4.0% (402)	-	2.0% (506)	-	1.5% (548)
[36] (USA; outcomes 1999–2000)	-	3.9% (795)	-	2.3% (690)	-	1.3% (972)
[39] (Canada)	-	6.0% (95)	-	1.3% (199)	-	-
[40] (The Netherlands)	-	3.4% (88)	-	0.8% (124)	-	-
Unpublished data (France)	-	7.6% (506)	-	-	-	-
[37] (USA; registry 2000–2001)	-	5.8% (482)	-	2.3% (650)	-	1.6% (936)
[54] (Spain)	-	-	10.1% (456)	-	-	-
[55] (Spain)	-	-	12.9% (827)	-	-	-
[41] (international study)	-	-	-	2.1% (285)	-	-
**Weighted mean rate**	18.4% (811)	5.6% (2864)	10.3% (2087)	2.0% (2767)	9.8% (123)	1.5% (2706)

Values are expressed as % (*n*). Hospitalization rates due to respiratory syncytial virus (RSV) in children ≤ 2 years old with bronchopulmonary dysplasia (BPD)/chronic lung disease (CLD), infants at gestational age 29–32 weeks without CLD, and infants at gestational age 32–35 weeks without CLD (combined analysis). -, not studied. Adapted from Simoes [44].
